# Dietary Supplements Potentially Target Plasma Glutathione Levels to Improve Cardiometabolic Health in Patients with Diabetes Mellitus: A Systematic Review of Randomized Clinical Trials

**DOI:** 10.3390/nu15040944

**Published:** 2023-02-14

**Authors:** Phiwayinkosi V. Dludla, Khanyisani Ziqubu, Sihle E. Mabhida, Sithandiwe E. Mazibuko-Mbeje, Sidney Hanser, Bongani B. Nkambule, Albertus K. Basson, Carmen Pheiffer, Luca Tiano, André P. Kengne

**Affiliations:** 1 Biomedical Research and Innovation Platform, South African Medical Research Council, Tygerberg 7505, South Africa; 2 Department of Biochemistry and Microbiology, University of Zululand, KwaDlangezwa 3880, South Africa; 3 Department of Biochemistry, North-West University, Mmabatho 2745, South Africa; 4 Department of Physiology and Environmental Health, University of Limpopo, Sovenga 0727, South Africa; 5 School of Laboratory Medicine and Medical Sciences, University of KwaZulu-Natal, Durban 4000, South Africa; 6 Centre for Cardio-Metabolic Research in Africa (CARMA), Division of Medical Physiology, University of Stellenbosch, Tygerberg 7505, South Africa; 7 Department of Obstetrics and Gynaecology, Faculty of Health Sciences, University of Pretoria, Pretoria 0001, South Africa; 8 Department of Life and Environmental Sciences, Polytechnic University of Marche, 60131 Ancona, Italy; 9 Non-Communicable Diseases Research Unit, South African Medical Research Council, Tygerberg 7505, South Africa; 10 Department of Medicine, University of Cape Town, Cape Town 7700, South Africa

**Keywords:** diabetes mellitus, cardiovascular diseases, cardiometabolic health, antioxidants, glutathione, inflammation, oxidative stress

## Abstract

Cardiovascular diseases (CVDs) continue to be the leading cause of death in people with diabetes mellitus. Severely suppressed intracellular antioxidant defenses, including low plasma glutathione (GSH) levels, are consistently linked with the pathological features of diabetes such as oxidative stress and inflammation. In fact, it has already been established that low plasma GSH levels are associated with increased risk of CVD in people with diabetes. Dietary supplements are widely used and may offer therapeutic benefits for people with diabetes at an increased risk of developing CVDs. However, such information remains to be thoroughly scrutinized. Hence, the current systematic review explored prominent search engines, including PubMed and Google Scholar, for updated literature from randomized clinical trials reporting on the effects of dietary supplements on plasma GSH levels in people with diabetes. Available evidence indicates that dietary supplements, such as coenzyme Q_10_, selenium, curcumin, omega-3 fatty acids, and vitamin E or D, may potentially improve cardiometabolic health in patients with diabetes. Such beneficial effects are related to enhancing plasma GSH levels and reducing cholesterol, including biomarkers of oxidative stress and inflammation. However, available evidence is very limited and additional clinical studies are still required to validate these findings, including resolving issues related to the bioavailability of these bioactive compounds.

## 1. Introduction

Diabetes mellitus is one of the prominent causes of death worldwide, with type 2 diabetes (T2D) responsible for a high proportion of premature casualties occurring at a rapid rate globally [1,2]. People with diabetes are known to be at an increased risk of developing cardiovascular diseases (CVDs) when compared with non-diabetic counterparts [3]. In fact, people with T2D are more likely to be obese and exhibit certain risk factors, such as elevated blood pressure, high cholesterol levels, and insulin resistance, which worsens their chances of having a heart attack or a stroke [3,4]. This explains an urgent need for treatments to improve cardiometabolic health or to alleviate risk factors that increase the likelihood of experiencing vascular events in patients with diabetes.

In recent years, there has been an increased use of dietary supplements to meet nutritional requirements [5,6]. It is currently understood that some nutritional requirements are not met through diet alone, hence the importance of some dietary supplements. These may come in many forms, including tablets, capsules, powder, gels, and liquids, that supply the body with essential vitamins and minerals [7,8]. It has long been postulated that oxidative stress, through enhanced oxygen-derived free radicals, may drive vascular damage [9]. Sustained exposure to hyperglycemia within a diabetic state can also have a profound effect in causing vascular damage through the detrimental effects of inflammation [10]. Both oxidative stress and inflammation are considered the major triggers for the development of CVD [11,12]. Dietary supplements have proven useful in replenishing intracellular antioxidants to neutralize oxidative damage and improve cardiometabolic health [13,14].

**Figure 1:**
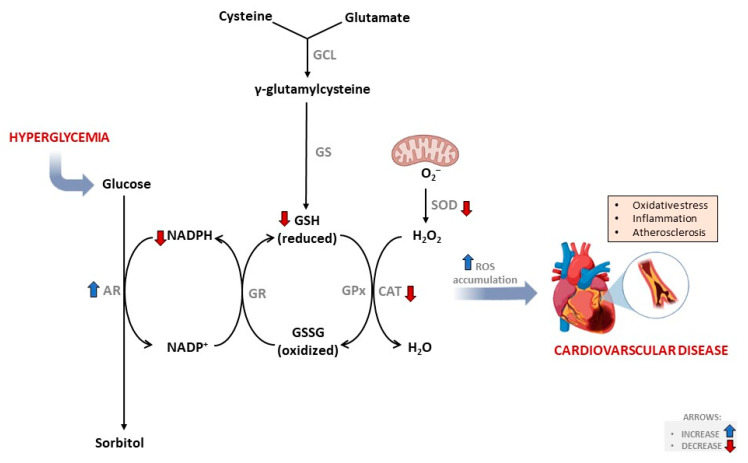
An overview of the glutathione biosynthesis pathway and its influence from external factors like free radical species. Briefly, the diabetic state is associated with depleted intracellular antioxidants (including glutathione) and increased risk of developing of atherosclerosis and cardiovascular disease. Both oxidative stress and inflammation are implicated in this process. Abbreviations; AR: aldose reductase, CAT: catalase, GCL: γ-glutamyl cysteine synthetase, GS: glutathione synthetase, GSH: glutathione, GR: glutathione reductase, GPx: glutathione peroxide, GSH: glutathione, GSSG: glutathione disulfide, NADPH: nicotinamide adenine dinucleotide phosphate, SOD, superoxide dismutase, ROS: reactive oxygen species.

Glutathione (GSH) is considered the major intracellular antioxidant within the human body, and its plasma levels range from 0.5 to 10 mmol/L [15]. In addition to its prominent role in protecting against cellular damage by detoxifying oxidative stress [16], GSH is fundamentally important for many intracellular processes, including cell proliferation, nutrient metabolism, and shielding against an undesired pro-inflammatory response [15,17]. Although humans can synthesize GSH, increased levels of oxidative stress, as seen in conditions of diabetes, can severely deplete its intracellular levels (Figure 1). In fact, reduced or depleted plasma levels of GSH have been linked with an increased risk for CVD, especially through atherosclerotic-driven complications [18,19]. Through reviewed evidence [20], it is increasingly becoming clear that enhancing the endogenous levels of GSH within the myocardium is vital to protect against oxidative stress-induced cellular damage. Our pivotal research has progressively shown that extracts or bioactive compounds from dietary sources like rooibos tea can protect heart cells against oxidative stress-induced damage by enhancing intracellular levels of GSH under the toxic conditions of hyperglycemia or diabetes [21,22,23]. However, information related to how dietary supplements modulate plasma GSH levels to impact cardiometabolic health in patients with diabetes remains relatively unknown, hence the importance of the current manuscript.

In this systematic review, we summarize and critically discuss available evidence on the potential role of dietary supplements in enhancing plasma GSH levels to improve cardiometabolic health in people with diabetes. Special focus is placed on evidence from randomized controlled trials (RCTs), with essential information on the effective dose and intervention, as well as the bioavailability profile of each included dietary supplement also covered.

## 2. Literature Search, Study Inclusion, and Quality Assessment

The systematic review was prepared following the Preferred Reporting Items for Systematic reviews and Meta-Analysis (PRISMA) guidelines (Supplementary file S1). The current study was not registered but the International Prospective Register of Systematic Reviews (PROSPERO) registry was comprehensively searched for any similar or ongoing studies to avoid duplicating systematic reviews on the same topic. Briefly, three investigators conducted a systematic search by accessing the major online databases/search engines like PubMed/MEDLINE and Google Scholar to identify qualifying RCTs. The search was conducted without any limitations, from inception to 30 December 2022. Relevant keywords and Medical Subject Headings (MeSH) terms included “glutathione”, “diabetes”, and “dietary supplements”. The current review only included RCTs reporting on the therapeutic link between the intake of dietary supplements and GSH levels in patients with diabetes. To allow the translational potential of the review, in vitro and in vivo studies were excluded but were briefly discussed within the Results section to expand on information concerning the therapeutic potential of each dietary supplement. The modified Downs and Black checklist (Supplementary file S2), which assessed the quality of evidence from randomized studies, was used to assess the quality of evidence, as previously described [14]. In fact, out of the 12 included RCTs, only 3 studies scored moderately [24,25,26], while the remaining literature presented with an acceptable quality of evidence (Supplementary file S2).

This systematic review was conducted to determine mainly whether supplementation with dietary compounds improves cardiometabolic health in people with diabetes. We included studies that met the following criteria.

Participants

Adult people (≥18 years) with diabetes mellitus.

Intervention

People with diabetes taking any dietary supplementation.

Comparator

People with diabetes (including those at increased risk of CVD) who received a placebo or who did not receive any dietary supplementation.

Outcome

The primary outcome was the regulation of plasma levels of GSH, including markers of oxidative stress and inflammation. Indicators or markers of cardiometabolic health were equally important and considered.

## 3. Results

### 3.1. Characteristic Features of the Included Studies

**Figure 2:**
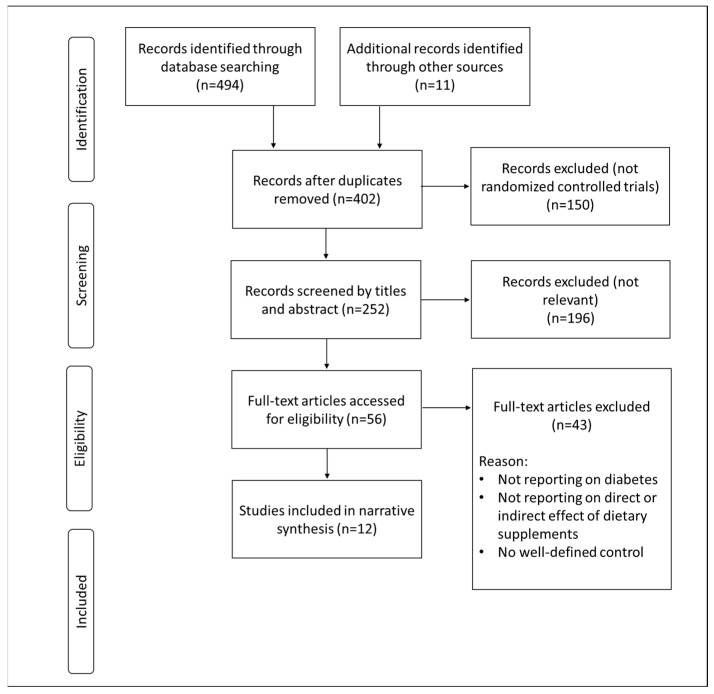
An overview of the flow diagram representing study inclusion.

A total of 505 records were recovered through the systematic search of PubMed/MEDLINE and Google Scholar (Figure 2). Most studies were excluded because they were not RCTs or irrelevant to the topic of interest. The retrieved studies were published between 1991 and 2022. Most studies emanated from Iran, while others were evenly spread between China, Brazil, Italy, Mexico, and Serbia. The study population included adult patients with an average age between 28 and 85 years. Most patients were obese, and some already had established CVD, especially coronary heart disease. Dietary supplements discussed include thiopronine, coenzyme Q_10_, (CoQ_10_), selenium, curcumin, omega-3 fatty acids, and vitamin E/D (Table 1). The discussed results encompass the relevant therapeutic evidence of each supplement (including effective dosages and treatment duration period), and its bioavailability profile.

### 3.2. Evidence on the Effects of Dietary Supplements on Plasma Glutathione Levels and Cardiometabolic Health

Table 1 gives an overview of evidence on the potential therapeutic effects of dietary supplements on enhancing plasma GSH levels to improve cardiometabolic heath in patients with diabetes. This includes studies reporting on the implication or regulation of markers of oxidative stress and inflammation. Apparently, it was evident as of 1991 [24] that administration of GSH or thiopronine, at doses reaching 6 and 12 mmol, could potentially lower blood pressure in hypertensive patients with diabetes. This was important to discover since it has already been established that plasma GSH levels are severely reduced in people with T2D [33]. Dietary intake of GSH does not directly translate to its enhanced plasma levels as many factors can affect its metabolism, thus leading to low bioavailability within the human body [34]. This has favored supplementation with GSH synthesizing molecules like N-acetyl cysteine that can enhance its endogenous levels, leading to reduced oxidative damage in conditions of hyperglycemia or hypertension [35,36]. Reduced bioavailability in response to its dietary intake better explains limited clinical studies that have directly evaluated the therapeutic effects of GSH supplementation in improving cardiometabolic risk profiles in patients with diabetes. However, this molecule has been shown to be well tolerated within the human body, while others argue that it can reach target tissues such as erythrocytes [37,38,39].

#### 3.2.1. Supplementation with CoQ_10_

Currently, there is limited information reporting on the therapeutic effects of thiopronine, but a few RCTs have reported on the potential impact of other dietary supplements like CoQ_10_, selenium, curcumin, omega-3 fatty acids, and vitamin E or D in enhancing GSH levels to improve cardiometabolic health (Table 1). For example, administration of CoQ_10_, at 100 mg daily for 2 months, could enhance plasma GSH levels while suppressing lipid peroxidation in overweight or obese patients with T2D and coronary heart disease [29]. Importantly, these positive effects are consistent with an improved metabolic profile, including serum insulin levels and Homeostatic Model Assessment for Insulin Resistance (HOMA-IR). Increasing evidence has accumulated on the beneficial effects of CoQ_10_ in increasing intracellular antioxidant responses to improve lipid profiles or cardiometabolic-risk factors in patients with diabetes [40,41,42,43,44]. More precisely, Zhao and colleagues conducted a meta-analysis showing that CoQ_10_ supplementation at doses of 100–200 mg/day was beneficial in reducing blood pressure in people with cardiometabolic diseases [45]. Zozina and co-workers reviewed evidence indicating that dosages of COQ_10_ vary in a wide range of 100–300 mg/day for it to be effective against CVDs [40]. However, it is stressed that little is known regarding the absorption of this molecule within the gastrointestinal tract and its amount in circulation after ingestion [40]. Therefore, because of its limited bioavailability, different formulations of CoQ_10_ supplements are increasingly being explored for their potential capacity to improve cardiometabolic health [46,47].

#### 3.2.2. Supplementation with Selenium

Administration of selenium, at an average dose of 200 µg/d for 3 months, was shown to improve insulin sensitivity and reduce cardiometabolic risk in patients with diabetes with or without congestive heart failure and coronary heart disease [30,31,48]. Importantly, such effects were consistent with effective modulation of the prominent markers of dyslipidemia, inflammation, and oxidative stress, including low-density lipoprotein (LDL)-cholesterol, and high sensitivity C-reactive protein (hs-CRP) and plasma GSH levels. Briefly, selenium is usually available in trace amounts within the human system and is considered a vital component that is required for many cellular functions. Explaining the importance of its availability in diet, selenium is essential for the optimal functioning of intracellular antioxidant enzymes including glutathione peroxidase (Gpx) and thioredoxin reductase [49]. Although available evidence supports the notion that optimal selenium status may improve cardiovascular health [30,31,48], it is often seen that external factors such as the mode of administration of this supplement may determine its therapeutic benefits [50]. Although dietary selenium is absorbed adequately, retention of its organic form is greater than that of the inorganic form [51]. There is also limited information on its quantification in food [52]. It has been indicated that selenium supplementation should not be encouraged in people already exposed to a high dietary intake because it may favor the development of insulin resistance [53], whereas others have shown that selenium intake can improve glycemic and lipidemic profiles in patients with T2D [54]. Overall, available evidence assures that more research is required to understand the therapeutic benefits of selenium against CVD-related complications.

#### 3.2.3. Supplementation with Melatonin and Curcumin

Melatonin and curcumin are some of the major dietary supplements that are progressively being explored for their potential health benefits. Melatonin is a ubiquitous element of the human diet and is also available as a health food supplement. Available evidence shows that administration of melatonin capsules at 10 mg daily for 3 months improved metabolic and lipid profiles by decreasing fasting plasma glucose concentrations, HOMA-IR, and total cholesterol levels in patients with T2D and coronary heart disease [55]. This effect was associated with lower blood pressure, and the reduction in markers of inflammation and oxidative stress like malondialdehyde (MDA), hs-CRP, and increased plasma GSH levels [56]. The potential benefits of melatonin in improving cardiometabolic health are associated with its strong antioxidant properties [57,58], while its biological properties extend to improving sleep patterns and reducing blood pressure in patients with hypertension [59]. On the other hand, the administration of curcumin at 1000 mg/day for 3 months could block lipid peroxidation by lowering MDA levels as well as enhancing plasma levels of GSH in patients with T2D and coronary heart disease [60]. Reviewed evidence indicates that regular intake of curcumin can improve glucose and lipid metabolism, attenuate inflammation, strengthen intracellular antioxidant response, enhance insulin signaling, and amend gut permeability in preclinical models of diabetes and CVD [61]. These findings confirm growing evidence supporting the potential benefits of curcumin supplementation in improving metabolic health and reducing CVD-related markers in patients with diabetes or related complications [61,62]. However, both melatonin and curcumin show poor oral bioavailability and large first-pass metabolism [63,64,65], with future research required to attempt to further investigate this phenomenon.

#### 3.2.4. Supplementation with Omega-3 Fatty Acids and Vitamin E/D

Foods or supplements rich in omega-3 fatty acids have also attracted considerable interest for their potential health benefits and are required for optimal metabolic health [66,67]. Fish and other seafood are recognizable sources of omega-3 fatty acids. Evidence presented in Table 1 shows that patients with T2D and coronary heart disease who received omega-3 fatty acids, at an average dose of 1000 mg twice a day for 3 months, had an improved metabolic profile, including decreasing insulin and hs-CRP concentrations, which were associated with enhancing plasma GSH levels [32]. It also remains important to determine whether different sources of omega-3 fatty acids, for example comparing fatty fish and vegetable oils, could provoke varied effects on glucose and lipid metabolism [68]. Available findings show that fish oil supplementation is more effective at reducing triglyceride levels, whilst marine and plant-based omega-3 fatty acids demonstrate more capacity in regulating glycolipid metabolism in patients with T2D [69]. However, a narrative review by Itsiopoulos and colleagues [70] argues that very limited information supports the beneficial effects of omega-3 fatty acids in improving metabolic parameters or reducing CVD-risk in conditions of diabetes. However, it is also evident that supplementation with omega-3 fatty acids can restore GSH levels and prevent oxidative damage in preclinical models [71]. However, available evidence related to its effects on cardiometabolic health in people with diabetes is still too limited to draw any conclusions.

Notably, fish is also the major source of vitamin D, while citrus fruits are considered the main supplier of vitamin E. There is increased interest in understanding the health benefits of supplementation with both vitamin D and E, especially concerning protection against cardiovascular complications [13,72]. The active metabolites of vitamin D include 25 hydroxyvitamin D and 1,25-dihydroxyvitamin D [73], with its predominant cytoprotective effects linked with protection against oxidative stress [74]. Reviewed evidence indicates that deficiency of vitamin D is associated with accelerated CVD-risk, which is mainly facilitated through defective autophagy as well as abnormal oxidant and inflammatory responses in some periclinal models [75]. This could explain results showing that vitamin D supplementation, at approximately 50,000 IU for 2 months, could improve metabolic profiles like fasting plasma glucose and insulin concentrations, and HOMA-IR in patients with T2D [76]. These effects are consistent with elevated plasma antioxidants like GSH, Gpx, and superoxide dismutase (SOD), as well as the reduced expression of markers of oxidative stress and inflammation, including MDA, 8-hydroxyguanosine (8-OHdG), and hs-CRP. Vitamin E also presents with strong cytoprotective effects in blocking oxidative stress [77]. There is already a clinical link between vitamin E intake and enhanced GSH levels in improving tissue glucose metabolism in patients with hypertension [78]. Although not directly affecting Gpx levels, administration of vitamin E at 450 mg for 3 months could improve blood glucose control, and enhance total antioxidant capacity and SOD levels in patients with diabetes and ischemic heart disease [25]. These findings indicate that, beyond their potential to enhance plasma GSH levels, these dietary supplements can affect a complex network of antioxidant mechanisms to improve metabolic health. Several factors can affect their bioavailability in different settings, and these include their interaction with other compounds as well as the host-connected factors, including the severity of a disease state [79,80]. However, very large RCTs are required to affirm any beneficial effects of these vitamin supplements.

#### 3.2.5. Supplementation with Plant Extracts

Emerging evidence has also looked at the potential therapeutic effects of different plant extracts, which are a rich source of diverse polyphenolic compounds with envisaged health benefits. Indeed, the evidence presented in Table 1 demonstrates that diabetic patients with chronic heart disease receiving a tablet of *Salvia miltiorrhiza* hydrophilic extract, at 5 g twice a day for 60 days, showed reduced markers of oxidative stress, including MDA levels and increased serum concentrations of GSH, SOD, and paraoxonase [26]. Similarly, patients with stable heart failure receiving Hershey’s extra dark 60% cacao chocolate and cocoa beverages containing 18 g of natural cocoa powder (twice daily) for 3 months showed enhanced GSH levels and reduced nitrotyrosilation and carbonylation of proteins within the skeletal muscle [27]. Furthermore, a diet containing granulated Brazil nut at 13 g per day for 3 months improved lipid profiles by reducing LDL-cholesterol while enhancing Gpx3 concentrations in hypertensive and dyslipidemic patients [28]. Although there is limited clinical evidence directly reporting GSH modulation, medicinal plant extracts, particularly polyphenols, can exhibit a wide range of biological effects, including ameliorative effects against oxidative stress and inflammation to improve cardiometabolic health [81,82].

## 4. Conclusions and Future Perspectives

**Figure 3:**
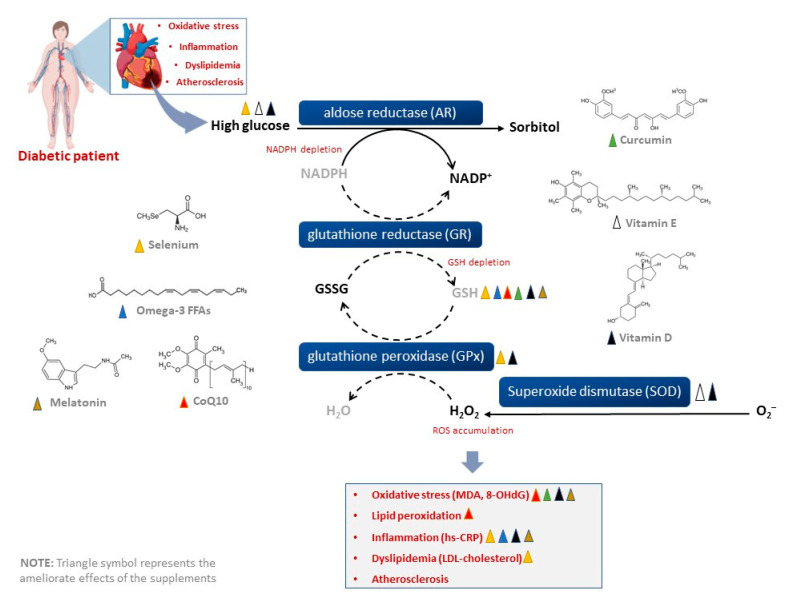
Dietary supplements such as coenzyme Q_10_, selenium, curcumin, omega-3 fatty acids, and vitamin E or D can potentially enhance intracellular antioxidants (including glutathione) to improve cardiometabolic health in diabetic patients. These effects are associated with reduced low-density lipoprotein-cholesterol, oxidative stress, and inflammation, while improving blood glucose control and blocking the destructive effects of sorbitol. Abbreviations; GSSG: glutathione disulfide, GSH: glutathione, MDA: malondialdehyde, 8-OHdG: 8-hydroxyguanosine, NADP(H): nicotinamide adenine dinucleotide phosphate, hs-CRP: high sensitivity C-reactive protein, LDL: low-density lipoprotein, ROS: reactive oxygen species.

A variety of dietary supplements with some biological properties to ameliorate metabolic complications are commercially available (including those discussed in the current review). The increased use of these products has created a need to understand their therapeutic potential beyond safety considerations [83,84]. Here, a systematic analysis of the literature revealed that the short-term intake of a variety of dietary supplements, including CoQ_10_, selenium, curcumin, omega-3 fatty acids, and vitamin E or D, can affect/stimulate plasma GSH levels and other antioxidants to improve cardiometabolic function (Figure 3). When assessed individually, these supplements may potentially improve cardiometabolic health by reducing blood pressure and LDL-cholesterol in patients with diabetes and even those with established CVD (Table 1). Because of their abundant antioxidant properties, the summarized evidence in Table 1 further suggests these dietary supplements can modulate prominent markers of inflammation and oxidative stress including hs-CRP and MDA. However, this review presents with many limitations and results should be interpreted with caution.

Notably, beyond the limited number, these studies do not report on the long-term therapeutic effects of these dietary supplements, and also included a relatively low number of participants. Importantly, larger sample sizes and more repeats are necessary to validate these results since it is already known that GSH-related genetic polymorphisms can affect disease pathogenesis and thus interfere with the patient response to treatment [85,86,87,88]. Furthermore, the generated information is mainly from one country (Iran) and does not represent an even geographic distribution of evidence. In addition, very little is known about the absorption and metabolism of these supplements, which is necessary to investigate their therapeutic potential. This may also explain the limited number of clinical trials showing the beneficial effects of these supplements against diabetes or CVDs. However, it remains important to explore this field of research further to discover novel therapies to alleviate CVD-related complications and prolong the lives of patients with diabetes mellitus.

## Figures and Tables

**Table 1:** Evidence on the effects of dietary supplements on plasma glutathione (GSH) levels and cardiometabolic health.

Author, Year	Country	Patients, Including Number and Average Age	Supplements, Dose and Intervention Period	Main Findings
Ceriello et al., 1991 [20]	Italy	Hypertensive and non-hypertensive diabetic patients (*n* = 20), with an age between 28 and 45 years	Received vitamin C, thiopronine and glutathione (GSH) at doses, reaching 6 and 12 mmol. Evaluation of blood pressure and heart rate was at 10/15 min intervals during a 1/2 h basal periods after intake of antioxidants	GSH and thiopronine displayed hypotensive effects at a dose of 12 mmol. However, all antioxidants had no effect on blood pressure in healthy normal subjects
Knezević et al., 2000 [21]	Serbia	Patients with ischemic heart disease (*n* = 51), and some with diabetes mellitus (18%), age not reported	Received tocopherol (vitamin E) acetate at 450 mg for 3 months	Treatment improved blood glucose control and superoxide dismutase (SOD) levels in patients with diabetes. However, increased total antioxidant capacity in patients without diabetes. No effect was seen with glutathione-peroxidase (Gpx)
Qian et al., 2012 [22]	China	Diabetic patients with chronic heart disease (*n* = 24), with an average age of 60 years	Received a tablet of *Salvia miltiorrhiza* hydrophilic extract at 5 g twice per day for 60 days in addition to their existing hypoglycemic therapy	Treatment did not affect lipid profiles but reduced markers of oxidative stress, including the levels of malondialdehyde (MDA). This was accompanied by increased serum GSH level, SOD, paraoxonase and GSH reductase
Ramirez-Sanchez et al., 2013 [23]	Mexico	Patients with stable New York Heart Association stages II and III HF and T2D (*n* = 5), with an age between 47 and 70 years	Received Hershey’s extra dark 60% Cacao chocolate and cocoa beverages containing 18 g of natural cocoa powder (twice daily) for 3 months, with a total of 100 mg (-)-epicatechin	Treatment promoted recovery in GSH levels and reduced nitrotyrosilation and carbonylation of proteins within the skeletal muscle of patients, as well as transcriptional factors related to the enhancement of SOD2 and catalase protein expression
Huguenin et al., 2015 [24]	Brazil	Hypertensive and dyslipidemic subjects (*n* = 52), with an average age of 62 years	Received a diet combined with granulated Brazil nut at 13 g per day (≈227.5 μg/day of selenium) for 3 months with 1 month washout interval	Treatment improved lipid profile by reducing low-density lipoprotein (LDL) levels and this was inversely linked with Gpx3 concentrations. However, did not affect the oxidative stress marker, 8-epi-prostaglandin F2alpha (8-epi PGF2α)
Raygan et al., 2016 [25]	Iran	Overweight or obese patients with T2D and coronary heart disease (*n* = 30), with an age between 40 and 85 years	Received 100 mg coenzyme Q_10_ (CoQ_10_) supplementation for 2 months	Treatment improved metabolic profiles, including serum insulin levels and Homeostatic Model Assessment for Insulin Resistance (HOMA-IR). Plasma total antioxidant capacity was neutralized after adjusting for age. However, GSH levels were increased, and MDA was reduced. However, treatment did not affect fasting plasma glucose, lipid concentrations and inflammatory markers
Raygan et al., 2018 [26,27]	Iran	Patients with congestive heart failure and impaired fasting plasma glucose (*n* = 26), with an average age between 45 and 85 years	Received selenium at 200 µg/d for 3 months	Treatment improved insulin metabolism and reduced cardiometabolic risk by decreasing LDL-cholesterol and hs-CRP, while increasing plasma total antioxidant capacity and total GSH levels
Raygan et al., 2019 [28]	Iran	Patients with T2D and coronary heart disease (*n* = 30), with an average age of 65 years	Received omega-3 fatty acids from fish oil at 1000 mg or omega-3 fatty acids from flaxseed oil at 1000 mg twice a day for 3 months	Treatment improved metabolic profile, including decreasing insulin and hs-CPR levels, while increasing the total antioxidant capacity, including GSH levels
Raygan et al., 2019 [29]	Iran	Patients with T2D and coronary heart disease (*n* = 27), with an average age of 65 years	Received selenium at 200 μg/day plus 8 × 109 CFU/day probiotic for 3 months	Treatment improved metabolic profiles such as fasting plasma glucose, and serum insulin levels. In fact, co-supplementation improved lipid profiles by reducing triglycerides, total cholesterol, and hs-CRP, while enhancing levels of nitric oxide, total antioxidant capacity and total GSH
Raygan et al., 2019 [30]	Iran	Patients with T2D and coronary heart disease (*n* = 27), with an average age of 65 years	Received melatonin capsules at 10 mg (2 melatonin capsules, 5 mg each) once a day for 3 months	Treatment improved metabolic and lipid profiles by decreasing fasting plasma glucose, HOMA-IR, and total cholesterol. In addition, reduced blood pressure and markers of inflammation and oxidative stress like MDA, hs-CRP and increasing plasma GSH levels
Shafabakhsh et al., 2020 [31]	Iran	Patients with T2D and coronary heart disease (*n* = 27), with an average age between 45 and 85 years	Received curcumin at 1000 mg/day for 3 months	Treatment improved sleep quality, by reducing Pittsburgh Sleep Quality Index. This was accompanied by reduced markers of oxidative stress like MDA. Furthermore, total antioxidant capacity and GSH levels were also increased. In addition, the expression of peroxisome proliferator-activated receptor gamma from mononuclear cells from peripheral blood was increased
Hoseini et al., 2022 [32]	Iran	Patients with T2D (*n* = 40), with an average age between 35 and 50 years	Received vitamin D at 50,000 IU for 2 months, with aerobic training program executed for 20–40 min/day, at 60–75% of heart rate maximum, for 3 days/week	Improved metabolic profile like fasting plasma glucose, insulin, and HOMA-IR; and reduced markers of oxidative stress (MDA and 8-OHdG). Markers of inflammation were also reduced, including hs-CRP, and gene expression levels of tumor necrosis factor-alpha (TNF-α), interleukin-1 beta (IL-1β), mitogen-activated protein kinases 1, nuclear factor kappa B (NF-κB) 1. This was consistent with elevated levels of total GSH, Gpx, SOD, catalase, and total antioxidant capacity

## Data Availability

Data regarding search strategy for study inclusion are available upon request from the corresponding author.

## Supplementary Materials

The following supporting information can be downloaded at: https://www.mdpi.com/article/10.3390/nu15040944/s1. Supplementary file S1: PRISMA checklist. Supplementary file S2: Quality assessment. Ref. [89] is also cited in Supplementary Materials.
